# Relationship between the density of degenerated neurons in the carotid body and changes in blood pH

**DOI:** 10.55730/1300-0144.5961

**Published:** 2024-12-12

**Authors:** Hakan KINA, Aydın Sinan APAYDIN

**Affiliations:** 1 Department of Neurosurgery, Faculty of Medicine, İstinye University, İstanbul, Turkiye; 2 Department of Neurosurgery, Faculty of Medicine, Karabük University, Karabük, Turkiye

**Keywords:** Blood pH, carotid body, glossopharyngeal nerve, subarachnoid hemorrhage

## Abstract

**Background and aim:**

The carotid artery is the main artery supplying blood to the brain. Carotid bodies (CB), located at the bifurcation of the carotid artery, regulate blood pH. This study aimed to assess the changes in CB-related pH levels following degeneration induced by ligation of both common carotid arteries (CCA).

**Materials and methods:**

We included 23 adult male New Zealand rabbits in the study. The animals were divided into three groups: a control group (n = 5), a sham group (saline injection, n = 5), and a study group subjected to bilateral CCA ligation (n = 13). pH levels and cardiac rhythms were monitored before, during, and after the procedure at two-day intervals over three weeks. Additionally, we quantified both healthy and degenerated neuron densities in the CB.

**Results:**

Initial pH levels were as follows: control group (7.42 ± 0.039), sham (7.39 ± 0.059), and study group (7.35 ± 0 .12). Prior to euthanasia, pH levels were the following: control (7.40 ± 0.029), sham (7.42 ± 0.041), and study (7.39 ± 0.12). The density of degenerated neurons in the CB was as follows: control (11 ± 4/mm^3^), sham (394 ± 74/mm^3^), and study (1363 ± 347/mm^3^). Statistical analysis showed the following significant differences: control vs. sham (p < 0.001), sham vs. study (p < 0.0005), and control vs. study (p < 0.000001).

**Conclusions:**

Increased degenerated neuron density in CB leads to reduced blood pH following CCA ligation due to acute ischemia of CB. Timely pH normalization occurs as vertebrobasilar reverse blood flow supplies CB, reversing neurodegeneration and normalizing pH.

## 1. Introduction

Carotid bodies (CB), located at the bifurcation of the carotid artery, receive blood from the external carotid arteries and serve as primary arterial chemoreceptors. They regulate cardiovascular and respiratory responses to hypoxia and maintain homeostasis. CB are primary chemosensitive, highly vascularized structures that regulate systemic circulatory and respiratory functions, maintaining homeostasis. Chemosensitive glomus cells, controlled by the glossopharyngeal nerve (GPN), react rapidly to changes in oxygen levels and blood acidity [1–3]. Sympathetic innervation is provided by the superior cervical ganglion (SCG), and sympathetic chemoreceptors within CB influence autonomic processes and glucose metabolism regulation [4]. Malfunction of CB disrupts respiratory, circulatory, and metabolic functions. Degeneration of CB neurons, particularly binuclear neurons (BN), can result in systemic pH imbalances, as observed after subarachnoid hemorrhage (SAH) [5]. BN, likely innervated by the vagus nerve (VN) or GPN, play a key role in pH regulation. Animal studies have shown that hypoxia-induced BN degeneration in CB correlates with lower pH levels and adverse outcomes. SAH may reduce BN density in parasympathetic regions, further exacerbating pH dysregulation [6]. This study evaluated the effects of CB degeneration caused by bilateral common carotid artery (CCA) ligation on systemic pH levels.

## 2. Materials and methods

We included 23 adult male New Zealand rabbits (2.5 ± 0.32 kg) in the study after obtaining approval from the Institutional Review Board on 05/04/2022 with protocol number E-10840098-772.02-2204. The rabbits were divided into three randomly assigned groups: a control group (Group A; n = 5); a sham group, given 0.5 mL of saline injected into the CB (Group B; n = 5); and the primary study group that underwent the CCA technique (Group C; n = 13). To minimize discomfort and reduce mortality risk, the rabbits were administered metamizole at 30 mg/kg body weight. Anesthesia was induced via isoflurane inhalation, followed by subcutaneous injection of 0.2 mL/kg of an anesthetic mixture consisting of 150 mg/1.5 mL Ketamine HCL, 30 mg/1.5 mL xylazine HCL, and 1 mL of distilled water before the surgical procedure. An additional 0.1 mL per kilogram of this mixture was given as needed during the procedure. All animals were placed in a supine position, and their anterior cervical areas were sterilized. A 3-cm incision was made in the middle of the neck, which allowed for the identification of the CCA, VN, jugular vein, and sympathetic chain on both sides. The bilateral carotid arteries were surgically separated and securely tied off using silk sutures. Postsurgery, the animals were observed for 3 weeks without medical intervention before euthanasia. CB were preserved in 10% formalin for 7 days, sectioned at 5 μm, and stained with hematoxylin & eosin and glial fibrillary acidic protein. Histological evaluations assessed neuronal health, and stereological techniques quantified neuron densities. The correlation between blood pH levels and deteriorated neuron density was also analyzed.

## 3. Results

**Figure 1:**
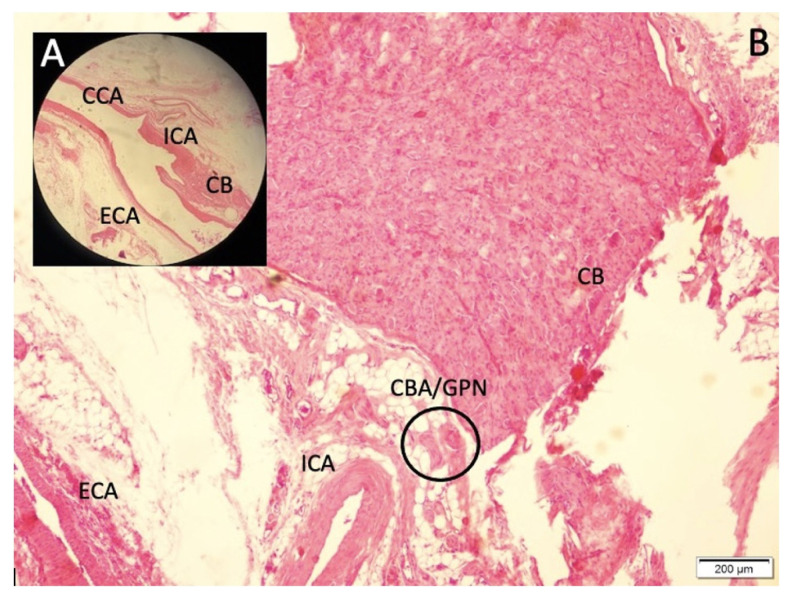
Magnified form with the demonstration of CB supplying artery-glossopharyngeal nerve fibers (CBA/GPN) with hematoxylin & eosin (H&E). A: common carotid artery (CCA), external carotid artery (ECA), internal carotid artery (ICA), and carotid bodies (CB)

**Figure 2:**
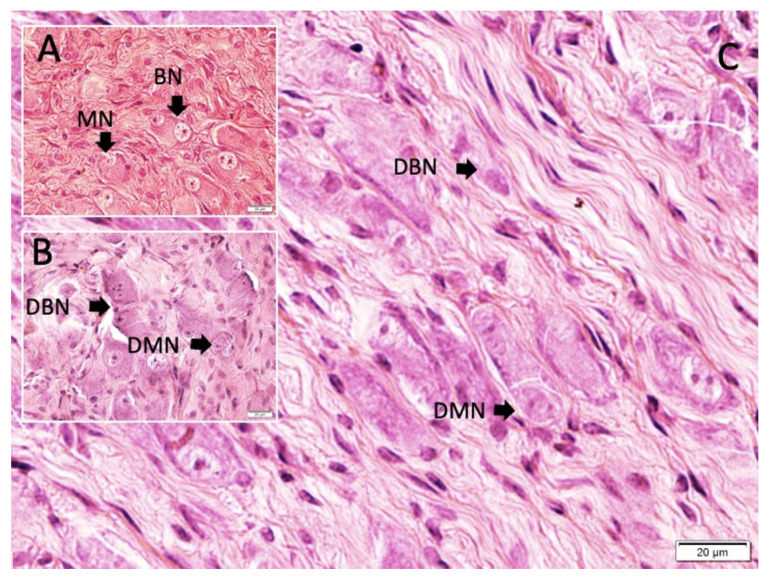
Histological appearances of the carotid body with H&E. A: binuclear neuron (BN) and mononuclear neurons (MN) in the control group; B: partially deformed binuclear neurons (DBN) and mononuclear neurons (DMN) in the sham group; C: significantly DBN and DMN in the study group.

**Figure 3:**
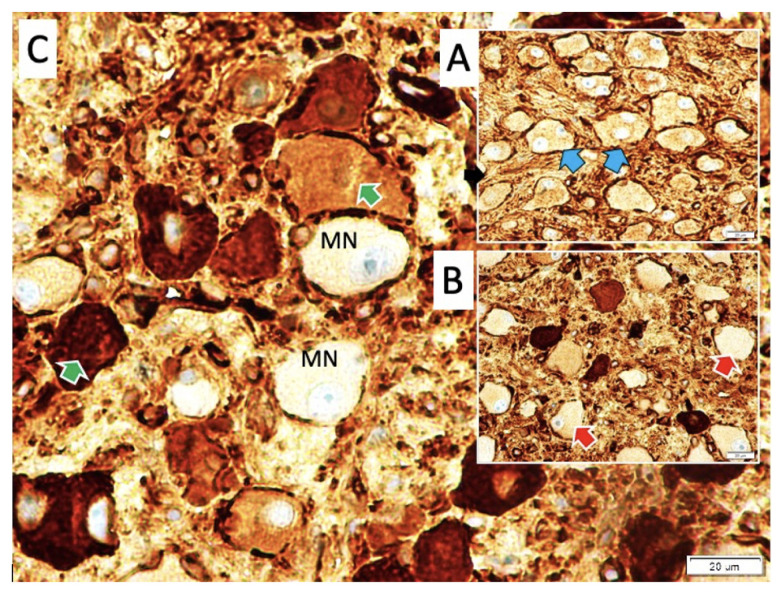
Histological appearances of the carotid body with glial fibrillary acidic protein. A: binuclear neurons (blue arrows) and mononuclear neurons (others) in the control group; B: partially deformed binuclear neurons (red arrows) and mononuclear neurons (others) in the sham group; C: significantly deformed binuclear neurons (green arrows) and mononuclear neurons (others) in the study group.

Common symptoms in the three surviving animals and during the premortem periods of those that did not survive included fever, apnea, altered consciousness, convulsions, respiratory disturbances, and cardiac arrhythmias. Postmortem anatomical examinations showed that the CB were typically located at the bifurcation of the CCA, near the internal carotid artery (ICA), and occasionally near the origins of the external carotid artery. The CB were oval-shaped, with an average diameter of 1.5 ± 0.24 mm (Figure 1). The SCG was located posterior to the CB, with extensions penetrating the superior poles of the CB, referred to as the sympathetic poles. The GPN and VN entered the CB from the posteromedial or lateral sides, designating these areas as parasympathetic poles. In animals exhibiting lower pH values, heart rhythm irregularities, respiratory issues, and thickened carotid arteries with reduced pulsatility were observed. Histopathological analysis revealed carotid plaques alongside a higher degree of CB degeneration and related disorders (Figure 2). Examination of the normal CCA structure assessed components such as the lumen diameter, inner elastic membrane, endothelial cells, vascular wall muscles, and adventitia. Observations included a slight reduction in the curvature of the inner elastic membrane, expansion of the inner surface and basilar artery, decreased wall thickness, and increased basilar artery volume. The experimental group displayed more pronounced CCA dilatation, elongation, convolution, and dolichoectatic configurations. Histopathological analysis, especially in the study group, revealed a flattening of the inner elastic membrane, thinning of the intima, shrinkage and desquamation of endothelial cells, lumen enlargement and dilation, and wall thinning following CCA ligation. The CB formed connections with GPN and VN at their larger ends, near the ICA and sympathetic fibers. Sympathetic nerve entry points into the CB were referred to as sympathetic poles, while parasympathetic nerve entry points were termed parasympathetic poles. Axonal bundles from GPN and sympathetic fibers were more numerous than those from VN. Ganglion cells were located in the peripheral layer of the CB. Our hypothesis suggests that most axons within CB are sensory, with a smaller proportion being nonsensory, significantly influencing sensitivity. A distinct bipolarity in the CB was identified, with parasympathetic/sympathetic poles and BN predominantly located in parasympathetic poles. These BN, likely connected through VN or GPN, were observed more in parasympathetic poles, contrasting with the predominance of sympathetic nerve fibers in sympathetic poles. A higher density of severely degenerated BN was noted in the parasympathetic poles compared to the sympathetic poles (Figure 3). Histological analysis revealed distinct appearances of the CB at parasympathetic and sympathetic poles, with close-up views showing mononuclear neurons (MN) and BN. BN were more prominent in parasympathetic poles, whereas MN were more evident in sympathetic poles. Degeneration of BN was more pronounced in parasympathetic poles, compared to the healthier appearance of MN and fewer BN in sympathetic poles. Initial pH levels across groups were as follows: control (7.42 ± 0.039), sham (7.39 ± 0.059), and study (7.35 ± 0.12). Before euthanasia, these levels were adjusted to the following: control (7.40 ± 0.029), sham (7.42 ± 0.041), and study (7.39 ± 0.12). The density of degenerated neurons in the CB was measured as control (11 ± 4/mm^3^), sham (394 ± 74/mm^3^), and study (1363 ± 347/mm^3^). Statistical analysis postligation showed significant differences between groups: control vs. sham (p < 0.001), sham vs. study (p < 0.0005), and control vs. study (p < 0.000001). Before euthanasia, the differences were the following: control vs. sham (p < 0.005), sham vs. study (p < 0.0001), and control vs. study (p < 0.00005).

## 4. Discussion

Acidosis is a critical complication of SAH, highlighting the CB network’s role in regulating systemic pH. Despite its importance, limited data exist on BN and their specific functions within CB. This study investigates the roles of MN and BN in CB in the context of acidosis following SAH. Although CB dysfunction and acidosis are well-documented, BN degeneration as a potential contributor to severe acidosis post-SAH is a novel finding. Gonzales pioneered histological techniques for studying CB structure, accurately identifying chemoreceptor cells in the glomus and their role in blood composition regulation [7]. Heymans et al. demonstrated that introducing compounds into the glomus alters heart rhythm via CB-mediated reflexes [8]. These reflexes regulate pH, respiratory frequency, heart rate, and arterial pressure. Degeneration of the CB-GPN network has been proposed as a mechanism for pH abnormalities after SAH [5]. Our findings show that BN are consistently present in all CB, with parasympathetic poles having higher BN density than sympathetic poles. After SAH, BN degeneration was more pronounced in MN-dominant sympathetic poles, suggesting a link between degeneration and impaired pH regulation. CB are primary chemosensitive, highly vascularized structures that regulate systemic circulatory and respiratory functions, maintaining homeostasis [9–11]. Located near the CCA bifurcation, CB predominantly receive blood from the external carotid arteries, occasionally from the internal carotid arteries [12]. Glomus cells in CB, coupled with GPN terminals, are highly sensitive to pH changes and can be activated by arteriovenous oxygen level variations of less than 2% [13]. The SCG provides sympathetic innervation to CB, and peripheral sympathetic chemoreceptors within CB regulate autonomic functions [14]. The sympathetic component of CB cells is crucial for glucose metabolism and transport [4]. Cerebrovascular dysfunctions can impair cerebral blood circulation, affecting the cardiovascular and respiratory systems [1]. Severe vasospasm following SAH can decrease cerebral blood flow, elevate intracranial pressure, lead to neuronal degeneration, and disrupt glucose metabolism, worsening the condition [8]. Timely revascularization of cerebral blood vessels effectively restores blood flow, minimizing brain injury and correcting pH imbalances caused by bilateral CCA ligation [15]. The correlation between neuron density and pH regulation during SAH suggests that CB dysfunction may contribute to these abnormalities. A study concluded that CB degradation decreases blood pH following SAH [16]. Our research investigated whether CB neuron degeneration affects pH regulation in SAH animal models. We identified a bipolar structure within the CB, with parasympathetic and sympathetic poles, and BN, predominantly in the parasympathetic poles. We hypothesize that BN, located in the parasympathetic poles, play a key role in dual innervation by the VN and GPN, which is critical for normal pH regulation. The presence of BN in CB is linked to sensitive CB nuclei, which may undergo fusion in response to hypoxia, either postnatally or genetically. BN formation has been documented in nervous tissues, such as dorsal root ganglia and cortical neurons of hypoxic rats [17,18]. Cellular fusion in the brain cortex following ischemic cerebrovascular diseases is considered adaptive, enhancing neuron functionality to better withstand hypoxia. In our study, SAH induced a significant increase in the density of degenerated BN in the parasympathetic poles of the CB. This degeneration exacerbated pH reduction and worsened the prognosis of SAH. We found significantly higher BN density in the parasympathetic fibers of the GPN at their entry points compared to the sympathetic fibers within the sympathetic poles. BN degeneration was more pronounced in subjects with lower blood pH, suggesting parasympathetic CB network injuries occur more frequently than sympathetic damage during SAH. These findings imply that decreased parasympathetic activity or increased sympathetic activity may contribute to acidosis in SAH. CB are closely connected to the GPN and VN at their larger ends and to sympathetic fibers near the ICA. Sympathetic nerves enter through the sympathetic pole, while parasympathetic fibers enter via the parasympathetic pole. Each nerve branch divides into multiple bundles upon entering CB, with GPN and sympathetic bundles more abundant than VN bundles. For instance, the sinus nerve has a length of 1.9 ± 0.4 mm and a diameter of 55 ± 12 μm, comprising approximately 254 ± 45 axons. A single blood vessel in the CB has a mean luminal diameter of 15 ± 3 μm at the medial capsular surface. Ganglion cells on the CB cortical surface show variable neuronal density, with nonmyelinated axons increasing distally in both poles. It is hypothesized that most axons in CB are sensory, with a smaller proportion being nonsensory, which is crucial for CB sensitivity. Our study identified a distinct bipolar structure in the CB, with parasympathetic poles showing a higher BN density than sympathetic poles. CB, primarily supplied by the external carotid artery, are essential for detecting arterial oxygen, carbon dioxide levels, blood pH, blood pressure, and glucose metabolism, thus regulating cerebrovascular and respiratory functions [19,20]. The fundamental unit of the CB consists of glomus cells adjacent to capillaries and connected to neurons in the PGN [2]. Activation of the PGN influences the paraventricular nucleus through CB neuronal networks [13]. Chronic hypoxemia may cause CB enlargement, and reduced blood flow triggers chemoreflex sensitivity due to changes in oxygen tension [21,22]. Dysfunction of the CB has been linked to cerebrovascular dysautonomia and panic disorder, with age-related impairments in CB function also reported [1,23]. Kanat et al. proposed that CB degeneration, particularly ischemic injury in the GPN, might contribute to decreased blood pH following SAH [5]. CB exhibit enhanced antiinflammatory properties postbirth, which may help maintain homeostasis and mitigate stressors such as hypoxia. Benzodiazepines affect respiration by enhancing GABAergic activity in the CB, influencing their role in respiratory regulation [24]. The peripheral chemoreflex pathway, involving the CB, PGN, and SCG, is mediated by cholinergic and purinergic neurotransmission, which is essential for regulating sympathetic activation and maintaining autonomic function [14]. Atherosclerotic changes in the carotid bifurcation can impair the baroreceptors and chemoreceptors in CB, leading to systemic sympathetic bias and autonomic dysfunction [1]. Cholinergic transmission from the paraventricular nucleus in the hypothalamus helps regulate plasma glucose levels via CB receptors [25]. Intrapulmonary airway chemoreceptors rely on CB glomus cell innervation to function effectively [26]. Hypoxia induces the formation of BN in various tissues, enhancing neuronal immunity, promoting reprogramming, and increasing the functional capacity of neurons to resist external stressors such as low oxygen levels. BN formation has been observed in the cortical area surrounding ischemic lesions, compensating for deficits caused by neuronal death after strokes [26]. These BN complexes are also found in the dorsal root ganglia, trigeminal ganglion, Purkinje neurons, and cervical ganglia, particularly as a response to stressors such as SAH. BN have even been noted in hepatocytes during adverse conditions. Binuclear complexes have been identified in the dorsal root ganglia, trigeminal ganglion, Purkinje neurons, cervical ganglia, and spinal cord following SAH, as well as in hepatocytes during stress or hazardous conditions [14,17,27,28]. The ganglionic cells of the autonomic nervous system, including both MN and BN neurons, have been detected in rabbits, particularly in the upper cervical ganglia and lactating mammary tissues. Binuclear motor neurons have also been found in monkey brains following trauma, while binuclear sensory neurons are frequently observed in neuronal cultures [29–32]. The current study provides comprehensive information on various neurochemical compounds found in CB. The nerve fibers surrounding blood vessels and the glomus cells in the chemoreceptive organ show immunoreactivity for several substances, including tyrosine hydroxylase, calcitonin gene-related peptide, substance P, galanin, vasoactive intestinal polypeptide, neuropeptide Y, calretinin, calbindin, parvalbumin, and nitric oxide synthase. The study explores the source and functional significance of these neurochemical compounds in the CB [33]. CB initiate defensive reflexes in response to hypoxia-induced hyperventilation [34]. Incomplete ischemic lesions have been observed in CB following bilateral common carotid ligation, leading to pH changes [35]. CB tumors are associated with baroreflex failure syndrome [36]. Chronic hypoxia results in structural changes, such as enlargement, hyperplasia of glomus cells, and neovascularization, and high-altitude conditions further stimulate CB activity [37,38]. Notably, the secretion of hypoxia-induced compounds from CB glomus cells is more pronounced in younger individuals, with a reduction in older age [39]. Ultimately, BN in CB are vital in efficient parasympathetic innervation, facilitated by the GPN and VN, which regulate critical physiological functions such as pH and metabolic processes. Dysfunction in these systems, particularly during events like SAH, could impair these vital functions and complicate treatment efforts [40,41]. Based on our results, the presence of BN cells in CB is essential for double parasympathetic innervation, maintained by the GPN and VN, which may regulate both pH (via GPN) and metabolic processes (via VN). If the CB/VN/GPN system collapses for any reason, dangerous disruptions in pH and metabolic regulation may become inevitable. These disturbances could be refractory to medication due to the potential damage to drug-carrying, mediating molecules and the coordinated neural pathways responsible for beneficial reflexes, particularly following SAH.

## 5. Conclusion

Our findings showed a higher concentration of BN in the parasympathetic poles of the CB, particularly where parasympathetic GPN fibers enter, compared to the sympathetic poles. Following SAH, we observed more significant BN degeneration in animals with lower blood pH, indicating that damage to the parasympathetic network of CB is more prevalent post-SAH. This suggests that decreased parasympathetic activity or increased sympathetic activity could play a significant role in acidosis during SAH. Our results support the theory that BN at the parasympathetic poles are crucial for the dual innervation from the GPN and VN, whereas sympathetic poles primarily receive input from the sympathetic nerves. The low neuron density in the CB observed in this study likely contributed to the significant decrease in blood pH seen in SAH, possibly impairing the metabolic processes essential for pH regulation and reducing the production of vasoactive neurotransmitters needed for glucose metabolism and cerebrovascular autoregulation. To mitigate the detrimental impact of SAH on the brain, exploring therapies aimed at protecting cerebral blood vessels may be beneficial. In ischemic conditions, CB neurons could fuse, and BN could be affected by hypoxia. Our results show that VN and GPN fibers are more susceptible to damage from SAH than sympathetic fibers, leading to more frequent BN denervation injuries. The literature suggests that sympathetic overactivity lowers pH while parasympathetic activity increases it. Ischemic degeneration of GPN and VN complexes can cause CB parasympathetic denervation and augmented sympathetic overactivity, decreasing pH. A high density of degenerated neurons in CB can lower blood pH after both CCA are compromised. However, when vertebrobasilar reverse blood flow begins, CB blood supply is restored, which can reverse neurodegeneration and normalize pH. This suggests that ischemic injury to the GPN-CB network may cause acidosis, which can be normalized by retrograde blood flow from the posterior circulation to the carotid vasculature.
